# Utilization of Partograph for Labor Management Among Healthcare Providers in Healthcare Facilities in India: A Systematic Review

**DOI:** 10.7759/cureus.44242

**Published:** 2023-08-28

**Authors:** Jamuna N, Mala Thayumanavan

**Affiliations:** 1 Nursing, Meenakshi Academy of Higher Education and Research (MAHER) University, Chennai, IND; 2 Management, Impetus Healthcare Skills Pvt Ltd, Chennai, IND; 3 Research, Meenakshi Academy of Higher Education and Research (MAHER) University, Chennai, IND

**Keywords:** attitude of healthcare providers, training on partograph, practice of healthcare providers, healthcare providers' knowledge, utilization of partograph

## Abstract

The partograph is a cost-effective tool used to monitor maternal and fetal conditions during labor. It plays a crucial role in facilitating prompt and appropriate intervention in the event of abnormal labor. Effective and timely detection of abnormal labor progression and proactive measures to prevent prolonged labor is crucial in mitigating the potential risks associated with postnatal hemorrhage, sepsis, obstructed labor, uterine rupture, and its subsequent complications. This study aims to conduct a comprehensive review to assess the level of knowledge, practice, and attitude related to the utilization of partographs among healthcare providers in the labor room of healthcare facilities. Systematic reviews of articles were collected from online databases to analyze the utilization of a partograph for labor management among healthcare providers in healthcare facilities in India. A total of 21 articles were selected to examine the outcomes of partograph utilization. The articles included healthcare facilities in 15 Indian states. The majority of the study participants were nurses and midwives. Most of the healthcare providers showed average or inadequate knowledge, while some demonstrated good knowledge about the partograph. Most articles concluded that there was inadequate practice and accuracy in completing the partograph. This inadequacy is observed across various designations of healthcare providers. This review found that the major challenges for healthcare providers include the unavailability of partographs, a shortage of healthcare providers, insufficient knowledge and competence, and limited training opportunities. Partograph training led to improvements in knowledge, tool utilization, and attitudes, as shown by significant differences in post-assessment outcomes. Improvement of healthcare in India necessitates strengthening partograph provision, ensuring adequate human resources, and providing regular training to healthcare providers. These interventions will serve to bridge the current gaps in partograph utilization in the country.

## Introduction and background

The partograph, commonly referred to as the partogram, is a graphical record that has been widely acknowledged and recognized as the foremost labor monitoring instrument across the globe. The World Health Organization (WHO) has recommended partograph usage during the active phase of labor as it aids in the timely detection of abnormal labor progress and facilitates immediate interventions, if necessary [1]. The primary objective of utilizing the partograph is to monitor the maternal and fetal conditions as well as the progress of labor [2]. WHO modified the partograph in 2000, focusing on labor progress, including cervical dilation, head descent, and contractions. The fetal condition is assessed by heart rate, amniotic fluid color, and fetal skull molding. Maternal condition is monitored by vital signs, urine output, urine tests for protein and acetone, drugs, IV fluids, and oxytocin administered during labor [3].

Partograph is a cost-effective instrument that aids healthcare professionals by utilizing it as an early warning system for enhancing decision-making in labor management [1]. Additionally, it plays a crucial role in facilitating prompt and appropriate intervention in the event of abnormal labor. Maternal and neonatal mortality could be reduced with access to high-quality maternal care throughout the perinatal period [4]. Early detection of abnormal labor progress using a partograph is important for preventing delays in managing labor complications and improving maternal and neonatal health outcomes [5]. Effective and timely detection of abnormal labor progression and proactive measures to prevent prolonged labor are crucial in mitigating the potential risks associated with postnatal hemorrhage, sepsis, obstructed labor, uterine rupture, and its subsequent complications [2].

Despite the partograph being used for 40 years, the persistence of mortality resulting from obstructed labor has raised concerns that the partograph is not reaching its potential in enabling the detection of deviations from normal labor and timely intervention [6]. Several research studies have been conducted in our nation to provide evidence regarding the utilization and challenges associated with partograph practices in India. The findings of the studies revealed that most healthcare providers possessed average to good knowledge regarding the partograph. However, their implementation of partograph practices was found to be insufficient [7-9]. A study that evaluated the partograph practices in 507 case records revealed that only 8.1% of the records included partograph plotting, and furthermore, most healthcare providers exhibited a negative attitude towards the utilization of the partograph [10].

Therefore, this study aims to conduct a comprehensive review to assess the level of knowledge, practice, and attitude related to the utilization of partographs among healthcare providers in labor rooms of healthcare facilities. It also studies the effectiveness of training on the utilization of partographs as well as the challenges associated with partograph plotting within healthcare facilities. It intends to serve as a guiding source for healthcare providers, with the goal of enhancing intrapartum monitoring using partographs to effectively save the lives of the mother and fetus.

## Review

Methodology

Data Sources and Searches

The evaluation of the utilization of partographs for the management of labor among healthcare providers in healthcare facilities in India was conducted in accordance with the standard Preferred Reporting Items for Systematic Review and Meta-Analysis Protocols (PRISMA-P 2020) checklist. The research articles were acquired by retrieving them from peer-reviewed journals through online databases like MEDLINE (PubMed), Google Scholar, Web of Science, Scopus, and Science Direct. This study encompasses scientific articles that have been published between January 2013 and May 2023.

The systematic execution of literature searches was conducted utilizing a comprehensive set of keywords such as “partograph”, “knowledge regarding partograph”, “practice on partograph”, “partograph utilization”, ‘healthcare providers’, “attitude towards partograph”, “challenges in partograph utilization”, “interventions to strengthen partograph use” and “training on partograph”.

The search terms utilized in this study were derived from the adopted PICO framework, which encompasses the identification of the problem, intervention, comparison or control, and desired outcome. This methodology was employed to ensure comprehensive access to all pertinent academic articles. The present systematic review excluded various forms of literature, including conference papers, notes, letters, and editorials. The articles analyzed in this review encompassed quantitative research designs, specifically cross-sectional studies, as well as pre-experimental and experimental studies focused on the utilization of the partograph.

Study Selection and Data Extraction

**Figure 1:**
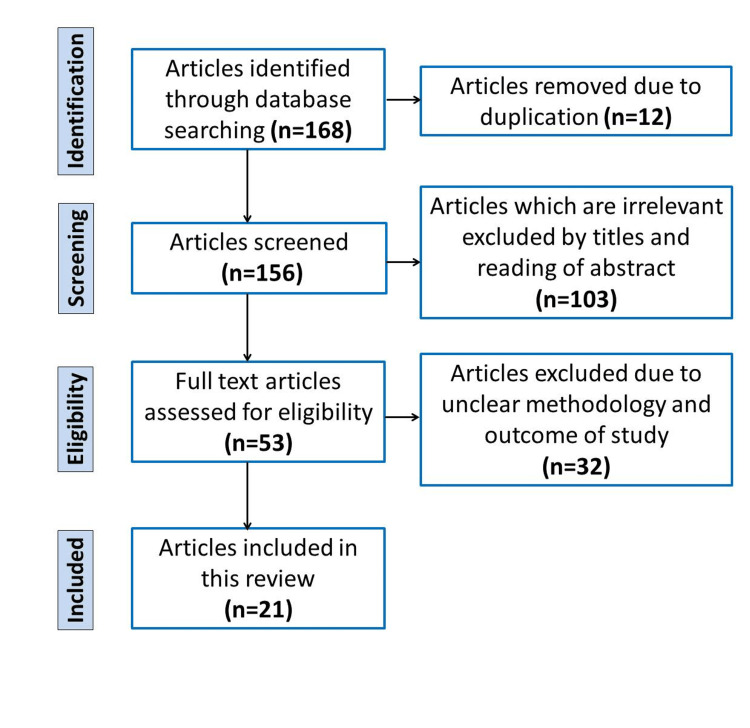
Flow diagram of the study selection process for a systematic review of the utilization of partograph

The employed search strategy yielded a collection of 168 articles sourced from diverse online databases; 12 duplicate articles were excluded. Out of the 156 articles that underwent screening, 53 full-text articles were meticulously evaluated to determine their eligibility. A total of 32 articles were excluded from the study due to unclear methodology and outcomes. In conclusion, a total of 21 articles that aligned with the intended study objective were selected for review to examine the outcomes of partograph utilization (Figure 1). To mitigate the risk of bias, a strategy was employed wherein two independent reviewers comprehensively extracted data and assessed the quality of the retrieved articles using criteria suggested by the Cochrane Handbook for Systematic Reviews of Interventions and the GRADE approach.

Results

Study Characteristics

The retrieved articles comprised a diverse selection of healthcare facilities across 15 states of India, namely Delhi, Punjab, Haryana, Himachal Pradesh, Jharkhand, Uttarakhand, Tamil Nadu, Gujarat, Maharashtra, West Bengal, Chandigarh, Odisha, Rajasthan, Madhya Pradesh, Karnataka, coastal regions of south India, and Puducherry. The retrieved articles studied were within healthcare facilities encompassing both the public and private sectors, with the larger portion being studied in public healthcare facilities. Most of the study participants were nurses and midwives. The studies comprised a wide range of sample sizes, consisting of a minimum of 30 participants and a maximum of 437 individuals. Table 1 displays the delineated characteristics of the retrieved articles.

**Table 1:** Characteristics of the study

Authors	Location of study	Study participants	Sample size
Harpreet Kaur et al., 2016 [7]	Ludhiana (Punjab)	Nurses	60
Soyam et al., 2022 [8]	Uttarakhand	Nurses	30
Singh S et al., 2017 [9]	New Delhi, Chandigarh, Vadodara and Meerut	Doctors (Interns, Junior residents and specialists)	150
Halder M et al., 2016 [10]	Malda (West Bengal)	Doctors and nurses	40
Palo SK et al., 2019 [11]	Kandhamal and Kalahandi districts (Odisha)	Health worker females, nurses and doctors	22
Kaur, 2019 [12]	Amritsar	Nurses	40
Pooja Bisht et al., 2022 [13]	Uttarakhand	Nurses	30
Singh S et al., 2018 [14]	Rajasthan, Gujarat, Odisha Uttar Pradesh, Tamil Nadu	Healthcare providers (Doctors and nurses)	1439 case records
Vidyashri et al., 2015 [15]	South India	Obstetric caregivers	502 case records
Chaturvedi S et al., 2015 [16]	Central Indian province of Madhya Pradesh	Nurse midwives	233
Jayabharathi et al., 2017 [17]	Karaikal district (Tamil Nadu)	Nurses	60
Sheela Asoo et al., 2021 [18]	Jodhpur	Nurses	60
Bajpayee D et al., 2020 [19]	Delhi, Punjab, Haryana, Himachal Pradesh, Jharkhand, Uttarakhand	Nurses	Baseline (427) End (194)
Gupta et al., 2020 [20]	Jodhpur	Skilled birth attendants	213
Yashoda Shrivastava, 2019 [21]	Indore	Nurses	80
Aradhana Chourasiya 2020 [22]	Bhopal	Nurses	40
Anuradha Rathore 2019 [23]	Greater Noida (Uttar Pradesh)	Nurses	40
Archa Prem et al., 2013 [24]	Mangalore	Nurses	30
Jinal Patel, 2020 [25]	Bhavnagar district Mehsana (Gujarat)	Midwives	30
Pratima et al., 2020 [26]	Pune	Nurses	40
Sharma et al., 2019 [27]	Haryana	Nurses	60

Utilization of Partographs Among Healthcare Providers

The retrieved 11 articles assessed the healthcare providers’ level of knowledge, practice, and attitude regarding the utilization of partographs. A total of nine articles assessed the practice of healthcare providers: six articles focused on knowledge and three articles on attitudes.

It was observed from Table 2 that, among the six articles reviewed, a major proportion of healthcare providers demonstrated average or inadequate knowledge [8,12,13] and good knowledge [7,9,16] in relation to the partograph. The analysis revealed that almost all reviewed articles consistently indicated a lack of appropriate practice and accuracy in completing the partograph. This inadequacy is observed across various designations of healthcare providers [7,8,10,11,14-17]. One study revealed that implementing a standardized partograph policy demonstrated notably improved partograph practices as compared to those operating without such routine protocols [9]. In relation to healthcare providers' attitudes towards the utilization of partographs, the majority of studies indicated a positive attitude [7,9], with only one study indicating a negative attitude [10].

**Table 2:** Utilization of partographs among healthcare providers

Authors	Study variables	Study findings
Harpreet Kaur et al., 2016 [7]	Knowledge, attitude and practices	The study participants had good knowledge (55%), a positive attitude (90%), and only a few practiced partograph (18.3%).
Soyam et al., 2022 [8]	Knowledge and practice	Most participants (53%) had average knowledge and inadequate practices (70%) for partograph utilization.
Singh S et al., 2017 [9]	Knowledge, attitude and practices	Participants had correct knowledge (60%–76%) and a positive attitude toward plotting the partograph. Participants from hospitals with a routine partograph policy had significantly better partograph practices compared to those without a routine policy.
Halder M et al., 2016 [10]	Practice and attitude	Documentation of partographs was found only in 8.1% of case sheets, with 59.9% of partographs being incorrect or incomplete. Participants had a negative attitude (67.5%) toward partograph monitoring.
Palo SK et al., 2019 [11]	Practice	The participants had done partograph plotting for 48.7% of deliveries, whereas only 1.03% of partographs were complete.
Kaur, 2019 [12]	Knowledge	Most of the participants (57.5%) had average knowledge, and 17.5% had poor knowledge regarding partographs.
Pooja Bisht et al., 2022 [13]	Knowledge	Most participants (59.0%) had inadequate knowledge of partograph monitoring.
Singh S et al., 2018 [14]	Practice	Plotting of partographs was observed in only 15.8% of deliveries. Vital parameters such as temperature, blood pressure, and pulse were never recorded for 19.3% of women.
Vidyashri et al., 2015 [15]	Practice	Participants’ practices towards plotting partograph parameters in terms of fetal heart rate (51.9%), cervical dilatation (48.8%), the descent of the presenting part (46.8%), molding (21.5%), membrane status (45.4%), and maternal blood pressure (34.8%)
Chaturvedi S et al., 2015 [16]	Practice	The partograph was used in only 6% of the reviewed records. Participants exhibited low utilization of partographs for monitoring labor and limited abilities to use partographs.
Geetha et al., 2015 [17]	Knowledge and practice	Participants had good knowledge (30%) and a poor level of knowledge (56.7%). About 50% of them had good practice, while 46.7% had average practice regarding the use of partographs.

Effectiveness of Training on the Utilization of Partograph

A systematic review of 10 articles aimed to evaluate the effectiveness of training interventions in enhancing healthcare providers' knowledge, practice, and attitude towards the utilization of partographs. Eight articles were analyzed to evaluate the practice of healthcare providers; nine articles assessed their knowledge, and two articles assessed their attitudes.

It was found that a greater proportion of healthcare providers had inadequate knowledge, practice, and attitude during the pre-assessment phase. The training program comprised various modes of instruction, namely on-the-job training, structured training, and self-instructional modules encompassing both individual and group-based approaches. The implementation of partograph training resulted in a notable improvement in participants' knowledge, utilization of the partograph tool, and attitudes, as evidenced by the statistically significant differences observed in post-assessment outcomes, which are presented in Table 3 [18-27]. 

**Table 3:** Effectiveness of training on the utilization of partograph

Authors	Study variables	Study findings
Sheela Asoo et al., 2021 [18]	Knowledge, attitude, and practice	In the pre-test, participants had inadequate knowledge (31.2%), a poor attitude (75%), and poor practice (85.9%). After training, knowledge increased to 76.72%, attitude to 50.86%, and practice to 79.02%.
Bajpayee D et al., 2020 [19]	Practice	The baseline assessment showed that 32% of participants fully completed partograph plotting. After the intervention, there was a significant increase to 81% in the end assessment.
Gupta MK et al., 2020 [20]	Knowledge and practice	Participants' mean knowledge before intervention was 13.04 and after intervention was 31.97. Partograph utilization increased from 35.2% to 73.2%.
Yashoda Shrivastava 2019 [21]	Knowledge and practice	A significant difference was found between pre- and post-test knowledge and practice after the implementation of training on the use of partograph.
Aradhana, 2020 [22]	Knowledge	Training increased the knowledge level of participants from 10.0% of average knowledge to 90.0% of good knowledge.
Anuradha, 2019 [23]	Knowledge and practice	Participants' knowledge increased from 50% to 80% in the post-test. Most participants increased their practice of partograph recording to 75.0% after training.
Archa Prem et al., 2013 [24]	Knowledge	The post-test knowledge mean (20.4) was higher than the participants’ pre-test knowledge mean was at 11.2 after the intervention.
Jinal Patel, 2020 [25]	Knowledge and practice	The participants’ pre-test knowledge mean was 12.1 and the practice mean was 4.47. Whereas in the post-test, the mean of knowledge increased to 23.03 and practice to 14.23 after training.
Pratima et al., 2020 [26]	Knowledge, attitude, and practice	In the pre-test, about 72.5% had poor knowledge, 30% had a moderate attitude, and 95% had poor practices. After training, a significant proportion of participants showed improvements in knowledge, practice, and attitude toward partograph use.
Sharma et al., 2019 [27]	Knowledge and practice	Participants' knowledge increased from inadequate (70%) in the pre-test to adequate (67%) in the post-test. In the pre-test, 65% had poor practice, while in the post-test, 78% had good practice in partograph plotting after training.

Challenges in the Utilization of Partograph

The identified challenges in the utilization of partographs encompassed multiple findings obtained from a comprehensive analysis of four scholarly articles. The foremost challenge entailed by healthcare providers was the unavailability of partographs [13,19,20], followed by shortages in healthcare providers [10,20], time-intensive processes [20], inadequate monitoring [13,19], insufficient knowledge and competence [13,19,20], limited training opportunities [13], and waste of time and effort [10].

Discussion

Maternal and neonatal mortality could be effectively prevented and managed if women had access to high-quality maternal care during the prenatal, intrapartum, and postnatal periods [4]. Prevention of adverse health outcomes associated with intrapartum complications can be achieved through early detection of abnormal labor progress using the partograph. Recognition and early detection of abnormal labor progress using a partograph can be the primary strategy for healthcare providers to prevent delays in the management of labor complications, which can lead to adverse maternal and neonatal health outcomes [5].

The WHO advocates for the utilization of the partograph in the assessment and documentation of maternal and fetal conditions as an essential tool for all women in labor [1]. However, the utilization of partographs was irregular and inconsistent across various settings and time periods, along with non-compliance with the recommended standards.

This research study encompassed a multitude of professional backgrounds, various populations, diverse measurements, and numerous states and districts within India. Most of the findings in this study indicated that healthcare providers possessed insufficient knowledge regarding the implementation of the partograph protocol. In a few studies, despite possessing good knowledge and maintaining a positive attitude, there was a noticeable lack of adherence to appropriate practices concerning the utilization of partographs [7,9,16].

The analyzed articles primarily focused on the utilization of partographs among nurses and midwives, who hold pivotal roles as frontline healthcare providers in intrapartum monitoring. The prevalent concern highlighted in these articles is the significant gaps in knowledge, practice, and attitude among nurse-midwives towards the utilization of partographs, and addressing these gaps is of utmost importance [8-17]. It has been proven in many studies that training and mentoring nurses increased their confidence to use and interpret partographs effectively [19,21-27]. The involvement of clinical mentors in monitoring and providing support has the potential to enhance the appropriate and consistent utilization of partographs by labor room nurses and midwives.

The research findings in one of the articles mentioned that the utilization of partographs in the monitoring of labor was observed to be insufficient among healthcare providers, primarily due to the reluctance or rigidity to modify behaviors and attitudes [11]. According to the findings of another study, it was suggested that a change in the negative attitude of healthcare providers through periodic training could potentially lead to increased utilization of the partograph, eventually leading to a reduction in intrapartum complications among women and newborns [9]. 

This review has identified that the foremost challenges or barriers among healthcare providers were the unavailability of partographs, a shortage of healthcare providers, insufficient knowledge and competence, and limited training opportunities [10,13,19,20]. A study suggested that the implementation of a hospital policy of routine plotting of partographs has the potential to enhance the utilization of partographs within tertiary care public hospitals in India [9].

This systematic review exhibited a multitude of notable strengths. A comprehensive search methodology was devised to encompass a substantial volume of scholarly literature pertaining to the utilization of partograph. The improvement of healthcare facilities in India necessitates bolstering the uninterrupted provision of partographs, ensuring adequate human resources in accordance with delivery demands, and providing periodic training and guidance to healthcare providers. These interventions will serve to bridge the current gaps in partograph utilization in the country.

## Conclusions

The findings from this study indicate that healthcare workers in most of the reviewed studies displayed inadequate knowledge, limited adherence to practice, and only moderately positive attitudes toward the utilization of partographs. Enhancing the knowledge, competency, and attitude of health workers towards the utilization of the partograph can significantly contribute to the improvement of accuracy in its completion. The outcome of this study demonstrated a pressing requirement for comprehensive reforms in the healthcare sector, particularly in terms of in-service training and continuous monitoring of service delivery across all tiers of healthcare facilities. These measures are crucial for enhancing compliance and accuracy in the utilization of partograph plotting, which, in turn, positively impacts maternal and perinatal health outcomes.

Recommendations

A) Strengthening healthcare facilities through the consistent provision of partographs stands as the initial measure to facilitate healthcare providers' efficacious implementation of partograph protocols. B) Continuous training and on-going professional development using effective instructional methodologies should be provided to every healthcare provider for better utilization of the partograph. C) Establishment of effective supportive supervision programs for healthcare providers, coupled with the enhancement of clinical proficiency in utilizing partographs, emerges as imperative elements for ensuring the long-term success of implementation efforts. D) Implementation of monitoring and auditing protocols for the partograph in clinical practice, encompassing aspects such as its completion, decision-making process, referral practices, and subsequent outcomes. 
